# Supplementary material for: Occurrence, Pathogenic Potential and Antimicrobial Resistance of Escherichia coli Isolated from Raw Milk Cheese Commercialized in Banat Region, Romania
Source: Antibiotics (Basel). 2022 May 27;11(6):721. doi: 10.3390/antibiotics11060721 (PMC9220297; doi:10.3390/antibiotics11060721)
Supplement: Supplementary file 1 [file antibiotics-11-00721-s001.zip › antibiotics-1735220-supplementary.pdf]

Table S1. Primers used for characterization of the isolated *E. coli* strains

Target gene	Primer sequences (5′–3′)	Size of amplicon (bp)	PCR conditions	Reference
16S rRNA	F: AGAGTTTGATCCTGGCTCAG<br>R: CTTGTGCGGGCCCCCGTCAATTC	1183	94 °C/3 min, 30 cycles (94 °C/30 s, 55 °C/30 s, 72 °C/1 min), 72 °C/10 min.	Magray et al., 2011 [30]
stx1	F: CGCTGAATGTCATTCGCTCTGC<br>R: CGTGGTATAGCTACTGTCACC	302	94 °C/2 min, 35 cycles (94 °C/1 min, 55 °C/1 min, 72 °C/1 min), 72 °C/5 min.	Blanco et al., 2004 [31]
stx2	F: CTTCGGTATCCTATTCCCGG<br>R: CTGCTGTGACAGTGACAAAACGC	516		

Table S2. The used minimum inhibitory concentration (MIC) breakpoints for the tested antimicrobials, in accordance with the correspondent references

Antimicrobial	MIC breakpoints (mg/L)		
	S ≤	R >	Reference
**Penicillins**			
Ampicillin	8	8	EUCAST[1]
Amoxicillin/clavulanic acid	8	8	EUCAST
Ticarcillin/clavulanic acid	8	16	EUCAST
**Aminoglycosides**			
Amikacin	8	8	EUCAST
Gentamicin	2	2	EUCAST
Neomycin	38	32	CA-SFM[2]
**Cephalosporins**			
Cefalexin	16	16	EUCAST
Cefotaxime	1	2	EUCAST
Ceftazidime	1	4	EUCAST
Cefepime	1	4	EUCAST
Cefalotin	8	32	CLSI[3]
Ceftiofur	2	8	CLSI-O
Cefquinome	2	8	CA-SFM
**Carbapenemes**			
Imipenem	2	4	EUCAST
Ertapenem	0.5	0.5	EUCAST
Meropenem	2	8	EUCAST
**Fluoroquinolones**			
Ciprofloxacin	0.25	0.5	EUCAST
Norfloxacin	0.5	0.5	EUCAST
Flumequine	4	16	CA-SFM
Enrofloxacin	0.5	4	CLSI-O
Marbofloxacin	1	4	CLSI-O
**Miscelaneous agents**			
Trimethoprim-sulfamethoxazole	40	80	EUCAST
Nitrofurantoin	64	64	EUCAST
Fosfomycin	64	256	CLSI
Piperacillin-tazobactam	16	128	CLSI
**Tetracyclines**			
Tetracycline	4	16	CLSI
**Amphenicols**			
Florfenicol	4	16	BMX VET

Legend: EUCAST - European Committee on Aantimicrobial Susceptibility Testing; CA-SFM - Comité de l'antibiogramme de la Société Française de Microbiologie; CLSI – Clinical and Laboratory Standards Institute; BMX VET – BioMérieux Veterinary;
